# Building stronger foundations: exploring a collaborative faculty mentoring workshop for in-depth growth

**DOI:** 10.1186/s12909-024-05775-7

**Published:** 2024-07-24

**Authors:** Rehana Rehman, Mahwish Arooj, Rahila Ali, Tazeen Saeed Ali, Kainat Javed, Saima Chaudhry

**Affiliations:** 1 Department of Biological and Biomedical sciences, Aga Khan University, Karachi, Pakistan; 2 Director Medical Education, Principal University College of Medicine and Dentistry, The University of Lahore, Lahore, Pakistan; 3 Department for Educational Development, Aga Khan University, Karachi, Pakistan; 4 School of Nursing and Midwifery, Aga Khan University, Karachi, Pakistan; 5 Department of Medical Education, University College of Medicine and Dentistry, The University of Lahore, Lahore, Pakistan; 6 CHPL, Faculty of Medicine and Dentistry, The University of Lahore, Lahore, Pakistan

**Keywords:** Faculty mentoring, Portfolio, Personal development, Quality education

## Abstract

**Background:**

Mentorship training programs demand a paradigm shift from theory-driven to hands-on practical approach with prioritization of preparation of mentors and mentees for their roles through self-awareness and targeted professional development planning. There is a lack of evidence generated from the health professions education institutions of global south regarding effectiveness of workshops in fostering mentorship culture.

**Methods:**

This mixed method study with convergent parallel design was conducted through a collaborative mentoring workshop; “Faculty Mentoring-Building stronger by digging deeper” by Aga khan University Medical College, Karachi and University of Lahore, Punjab, Pakistan. Objective of the research was to emphasize the importance of faculty mentoring program, roles and responsibilities of mentors and mentees and perception of the participants regarding the associated role of institutions. It aimed to educate faculty members to develop personal development plans for becoming effective mentors and mentees. The demographic data was collected before the workshop, during the workshop data was collected from SWOT analysis, followed by goal settings and the action plans made by participants at the end. Post workshop online feedback was acquired by a questionnaire to comprehend participants’ educational attainment. Association between quantitative findings was done through ANOVA, while the qualitative data was subjected to thematic analysis.

**Results:**

Total of 37 faculty members participated in the hands-on workshop. All faculty equally perceived the workshop as satisfactory and reported that hands-on practice led to positive experience of setting clear goals and action plans in developing oneself both as mentor and mentee. Themes identified were; Faculty Mentorship Program, Personal development Plan of Mentors and Mentees and Building Positive Mentor-Mentee Relationships. Voluntary structured program, choice of more than one faculty mentor and portfolio development based on personal SWOT was recommended by participants for the success of formal mentoring programs.

**Conclusion:**

Medical Faculty of Pakistani Universities at all career levels is interested in development of formal mentoring programs in their universities. Formal training for the same is also recommended by the participants. Institutions should cultivate a culture of mentorship that supports the professional growth and success of academics for cultivating the minds that are in turn shaping our future generations.

## Introduction

Mentoring is considered as reciprocal learning relationship between mentor and a mentee which has an impact on teaching, research and teacher education programs [1]. Faculty members though continue to gain experience and skills through observation of self and knowledgeable others, yet getting formally mentored both at a professional and personal level is shown to add to increased efficiency and promote skills [2]. By implementing effective mentoring programs, institutions can improve the professional development and job satisfaction of their faculty members, which can lead to improved research productivity, faculty satisfaction and overall institutional success. It is need of the day that institutions should organize sustainable faculty mentorship program to cater career development, provide psychosocial support and instill professional development in the faculty [3].

Foundation of these programs was laid down in the late twentieth century yet there still is a dearth of formal mentoring programs in the academic institutions of developing countries and many institutions are struggling for their initiation and sustainability [4]. Limited information and characterization of programs to provide professional mentoring and development opportunities for junior faculty members contribute to the challenges in designing effective mentorship programs especially in low- and middle-income countries [5, 6]. In addition to that the current state of faculty mentorship lacks standardized practices, leaving mentorship relationships largely informal and inconsistent hindering their ability to navigate challenges related to teaching, research, and professional advancement. Additionally, feedback mechanisms and assessment to improve mentoring in academia are limited [7]. On the contrary, the rapid evolution of educational methodologies, technological advancements, and changing student demographics require faculty members to continually adapt and innovate. The need for continuous formal mentorship training to improve career development resource for junior faculty becomes even more important today [8].

Mentorship training programs demand a paradigm shift from theory-driven to hands-on practical approach with prioritization of preparation of mentors for their roles to educate and train the mentees [9]. One of the consistent approaches for enhancing mentoring effectiveness includes longitudinal mentoring training programs, workshops and courses [10]. These include forward-thinking faculty development workshops with description of the action plans of individual participants, practices, barriers to mentoring, post-workshop survey evaluations and follow-up actions to establish local mentoring programs and practices [10, 11]. Mentorship workshops provide structured training for mentors, improve their competencies in working with diverse group of mentees which implies that number of mentor training interventions can be designed to strengthen the involvement and perseverance of mentees towards their goals and career intentions [10, 11]. The trainings can be augmented with distribution of hand-outs and acceptance of the mentoring process with pride by the leadership for enrichment of mentoring culture [10]. A study identified mentoring workshops as one of the strategies to to acquire integrated mental health care in low- and middle-income countries [12]. In Nepal, mentorship helped to facilitate post-partum family planning, its institutionalization and use of postpartum intrauterine device services [13]. These workshops can be cost-effective and accessible options for educating mentors and enhance mentoring competency in faculty, staff, and trainees [14]. The mentorship programs can therefore be developed, evolved and implemented through feedback obtained from the workshops and also help to dissipate myths and provide meaningful communication and contact between mentors and mentees.

With reference to current mentoring culture at Aga Khan university Pakistan (AKU), informal faculty mentoring has been practiced since the time of inception, similarly the University of Lahore Pakistan (UOL) is running the program informally since 2015. However, a standardized faculty mentorship forum was implemented in AKU in 2019 to create a supportive learning environment and enhance the capacity of faculty mentors [15, 16]. Qualitative investigations during course of the program highlighted the need for continuous improvement of mentoring practices, for strong foundations and rewarding culture of mentoring [17, 18]. After identifying the need a series of workshops for long-term career accomplishments of mentees and improvement in the mentorship programs; as is suggested by the literature [19].

However single institution practices need to be validated in multi-institutional contexts. Collaborative workshops can compensate for resource shortages and facilitate the achievement of maximum outcomes [20]. Post-workshop analysis in can provide insights into the program, identify necessary changes in program design, offer guidelines for mentor preparation, evaluate career satisfaction outcomes across institutions [21]. Hence a workshop was designed for addressing two institutions; one having a formal structure (AKU) and other (UOL) running the program informally to explore to emphasize on the importance and benefits of faculty mentoring program, roles and responsibilities of mentors and mentees and collect perception of the participants regarding the role of faculty mentoring programs of medical academics in these institutions of global south.

The answers were sought for the perceived importance and benefits of faculty mentoring programs, their perception of their own roles and responsibilities and that of the institution, the realization of the importance of mentor mentee relationship and value of self-awareness through personal analysis and development of goals and action plans for becoming a successful mentor or a mentee.

## Methods

This mixed method study with a convergent parallel design was conducted through the implementation of a faculty mentoring workshop during May till December 2023, with the medical faculty of AKU and UOLn after approval from Ethical Review Board of AKU; ERC :2021-6127-17832. Informed consent was obtained from all participants to use anonymized activity data collected before and during the workshop and feedback data both quantitative and qualitative at the end of the workshop for publication. The unique protocol ID submitted to clinicaltrials.gov is 2021-6127-17832.

### Background and organizers of the workshop

The Aga Khan University Karachi, Sindh and The University of Lahore, Punjab Pakistan are the two high ranked universities in the country imparting quality medical education [22]. Both institutions have the most sought-after faculty in the field of medicine. The formal faculty mentoring program was introduced in AKU in 2019 while this program runs informally at the University of Lahore. The organizers of the workshop from AKU were its faculty mentoring chair and from UOL were the faculty development program lead. There were two facilitators for each workshop. Same facilitators conducted the workshop at both institutions. Both the facilitators (MA and SC) were Professors and along with PhD’s in their fields had a Master’s degree in medical education and more than ten years of experience in conducting the workshops in the field of health professions education.

### Details of the workshop

#### Designing and facilitation

Faculty mentoring workshop with the title “Faculty Mentoring; building stronger by digging deeper” was designed as a two-day workshop of three hours each for the faculty members of the two institutions at separate times.

#### Workshop learning outcomes and activities

The learning outcomes of the workshop day 1 were, importance and benefits of faculty mentoring program, roles and responsibilities of mentors and mentees, the ethics and stages of any mentoring relationship. The activities included group discussions and ethical dilemmas as demonstrated by role plays by the participants in groups of three. Day 2 agenda was developing a mentor or a mentee portfolio with special focus on self. The activities were personal Strength, Weakness, Opportunities and Threats (SWOT) analysis, goalsetting for a mentor and a mentee and developing an action plan. Most of the day was dedicated to hands-on individual activities followed by voluntary sharing of the personal development goals and a discussion on the correct ways of framing goals and realistic ways of writing an actionable plan with milestones for tracking success.

### Data collection

#### Data sources

Qualitative Data:

- During Workshop: SWOT analysis, goals set and the action plan developed by each participant during the group activities and the individual hands-on sessions the workshop.
- Post workshop: Reflections of participants regarding; the three things that they learnt, the questions still in their mind and one change that they will make after the workshop in themselves for being effective mentor or mentee.

Quantitative data.

- Pre workshop: The data of the faculty was collected before the workshop in terms of their title and years of experience.
- Post workshop: feedback on the workshop’s effectiveness was acquired by a questionnaire which was developed by department of Continuous Medical Education at Aga Khan University (Appendix A). The content, construct and criterion validity were checked before pilot testing by subject matter experts and it was ensured that questions were relevant to the objectives of the activity and was clear and understandable to the respondents. Responses were further categorized into levels of satisfaction: “Unsatisfactory,” “Satisfactory,” and “Excellent.”.

### Data analysis

The data collected before during and after the workshop was segregated for quantitative and qualitative analysis based on the study design. The quantitative data was coded and entered in IBM SPSS version 29. The level of satisfaction, perceived effectiveness of the training on the competency levels, and the career level of the faculty were computed as frequencies and percentages. ANOVA test of significance was used to compare the level of satisfaction of the workshop and the career level of the faculty. For inferential statistics the p-value of less than 0.05 was taken as significant keeping the confidence level at 95%.

Thematic analysis of all the per workshop and post workshop qualitative data was done through the inductive approach as there were no preconceptions involved in collecting or analyzing the collected data. The qualitative data was categorized and coded by two authors independent of each other. The codes were taken as the statements written by the faculty members during and after the workshop. After the first phase of coding, it was reviewed by all authors and subthemes and themes were generated. The authors carried out content analysis of the text. Responses of all categories were collated.

## Results

The study was based on the hands-on workshop with voluntary participation in which 16 faculty members joined in the first workshop at AKU while 21 joined in the second workshop conducted at UOL. Data from all these 37 participants were included in the study. Faculty members in their different academic ladder; early-Career: Instructors and Senior Instructors, Mid-Career: Assistant Professors, Seniors: Associate Professors and Professors/Consultants participated in the workshop.

### Quantitative findings

**Fig. 1:**
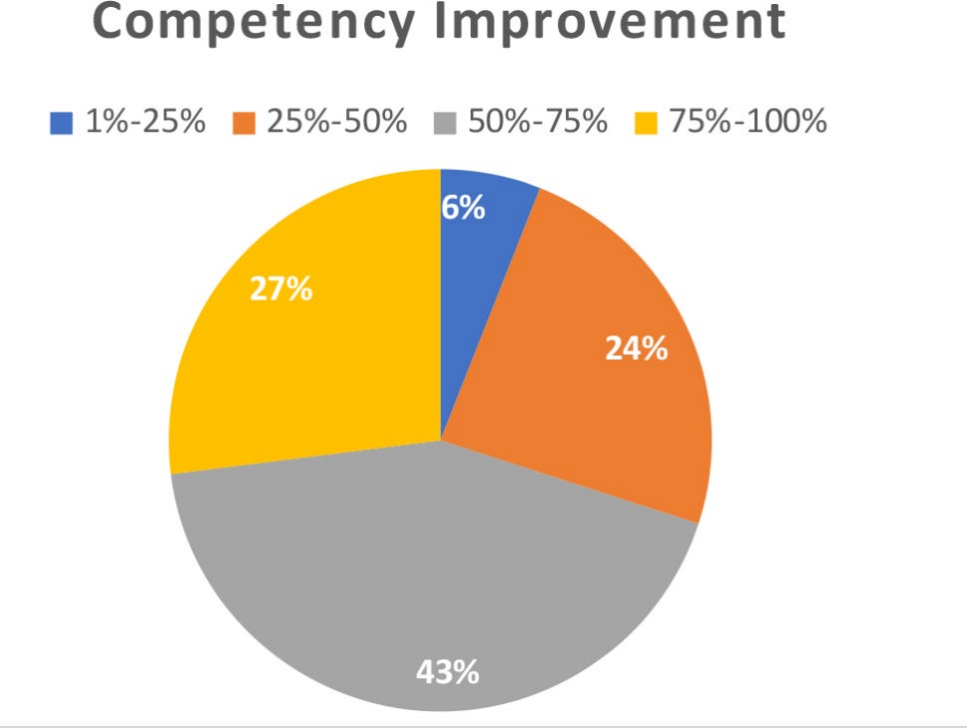
Perceived competency improvement post mentoring workshop by medical faculty

As shown in Table 1, all the participants were satisfied regarding the completion of workshop objectives, the level of the content delivered, time management, learning something new and overall rating. All of them were of the opinion that they will be happy to recommend this workshop to their peers. One of the participants could not get the satisfactory answer to the queries and one was of the view that knowledge gained was just a refresher, however majority of participants (62.2%) regarded this activity as an excellent resource for clarifying their roles and contributions towards faculty mentorship program. Another question asked after the workshop was the effect of this workshop on perceived level of increase in competence of the attendees as faculty mentors and mentees in which 70% of the participants documented an improvement ranging from 50% to 100% (Fig. 1).

**Table 1:** Participants response on effectiveness of workshop

Sr	Survey Questions	Responses *n* (%)		
		Unsatisfactory	Satisfactory	Excellent
1	Objectives of the activity Achieved	-	13 (35.1)	24 (64.9)
2	Presentations at the participant’s level of understanding	-	12 (32.4)	25 (67.6)
3	Acquired new Knowledge	1 (2.7)	12 (32.4)	24 (64.9)
4	Time Management		11 (29.7)	26 (70.3)
5	Queries responded	1 (2.7)	11 (29.7)	25 (67.6)
6	Relative to where you were prior to participating in this activity, please rate how well this activity has affected your ability to understand the topic/subject?	-	17 (45.9)	20 (54.1)
7	Based on your participation today, how will you rate this activity as a recommendation to your peer/colleagues?	-	16 (43.2)	21 (56.8)
8	Overall assessment of the activity	-	14 (37.8)	23 (62.2)

bivariate analysis was conducted between the career levels and the questions of knowledge acquisition to explore whether the faculty at different levels of their career have similar level of knowledge regarding the formal faculty mentoring or is there a difference. Table 2 presents participant feedback that is categorized into three aspects: “Acquired New Knowledge,” “Improved Understanding of the Topic,” and “Competency Improvement”. The results indicate that there are no statistically significant differences in participant feedback among different career levels for all three competency aspects suggesting that the activity’s effectiveness was consistent across early-career, mid-career, and senior faculty members and all tiers of faculty equally benefited from the training.

**Table 2:** Association between career level and knowledge acquisition

Career Level*	Acquired New Knowledge n (%)						Level of Significance
	Unsatisfactory		Satisfactory		Excellent		
Early-Career	-		1 (10)		9 (90)		0.061
Mid-Career	-		6 (42.9)		8 (57.1)		
Seniors	1 (7.7)		5 (38.5)		7 (53.8)		
	**Improved understanding of the topic** n (%)						
	Unsatisfactory		Satisfactory		Excellent		
Early-Career	-		4 (40)		6 (60)		0.875
Mid-Career	-		8 (57.1)		6 (42.9)		
Seniors	-		5 (38.5)		8 (61.5)		
	**Competency Improvement** n (%)						
	1-25%	25-50%		50-75%		75-100%	
Early-Career	1 (10)	3 (30)		3 (30)		3 (30)	0.953
Mid-Career	1 (7.1)	2 (14.3)		6 (42.9)		5 (35.7)	
Seniors	-	4 (30.8)		7 (53.8)		2 (15.4)	

Early-Career: Instructors and Senior Instructors, Mid-Career: Assistant Professors, Seniors: Associate Professors and Professors/Consultants

### Qualitative findings

Three major themes identified were identified related to system factors, social factors and personal factors as listed in Table 3. Themes identified are; (1) Faculty Mentorship Program with a focus on the structure and the components of the program from the organizational and institutional perspective, (2) Personal development plan of mentors and mentees with a focus on self-development, training, personal qualities and competencies and (3) Building Positive Mentor-Mentee Relationships with a focus on rapport building, culture of trust, mentor mentee communication and compatibility.

**Table 3:** Themes, subthemes and codes from the discussion and feedback of faculty mentoring workshop

Theme	Subthemes	Code
Faculty Mentorship Program	Structure of the Program	Formal mentorship
		Establishing differences between mentoring and hierarchical relationships
		Suitability in the cultural context
		Mentorship at different career levels
		Identifying qualities of effective mentors and mentees
		Assessing how the program meets personal and professional needs
		Arranging extracurricular activities for faculty and student mentees
	Organization of mentorship support	Embracing the idea of having more than two mentors
		Changing expectations based on learning from multiple mentors
		Valuing perspectives from different mentors
		Fostering a culture of helping others and providing equal opportunities for growth
		Allowing mentees to change mentors if not satisfied with the current one
		Solving personal and professional challenges faced by mentees
	Preparing/ developing/skill development of mentors and mentees	Ethics and responsibility of mentorships
		Integrating workshops and skill development for faculty and mentors
		Utilizing workshops to improve mentoring skills and subject expertise
		Evaluating effectiveness of mentors and mentees
		Evaluating success and impact of training sessions in mentorship programs
		Implementing mentoring at the department level
		Importance of feedback and improvement in mentorship programs
		Looking for new learning opportunities in different situations
		Teaching others and sharing knowledge with small groups
	Organizational Support:	Ensuring senior management support for mentorship program
		Utilizing portfolios as part of appraisals for promotion/incentives
		Awareness and knowledge of faculty mentoring
		Implementing faculty mentorship in an academic setting
Personal development Plan of Mentors and Mentees:	Self-assessment	Self-assessment through SWOT analysis
		Self-improvement through mentoring
		Identifying weaknesses
		Recognizing and respecting oneself
		Developing skills through mentorship
		Overcoming challenges in personal and professional development
		Building a strong personality and self-belief for effective mentoring
		Encouraging mentees to do SWOT analysis
		Non-judgmental self-assessment and avoiding negative self-analysis
	Personal Development & Leadership	Taking clear decisions and reducing confusion
		Pushing oneself to improve and rewire negative motivation
		Identifying weaknesses and working on self-improvement
		Learning to love oneself personally and professionally
		Taking ownership of one’s success and goals
		Leading one’s way with guidance from mentors
		Empowering oneself through mentorship
		Changing perspectives and embracing change for personal growth
	Goal Setting	Writing down goals for clarity and accountability
		Develop goals
		Updating knowledge and seeking new learning opportunities for development
		Defining clear expectations from the program
		Building evidence-based study for formal mentorship programs
		GROW criteria for goal setting
		Setting short-term goals
		Convincing and convincing others about goals
	Action Planning	Addressing time management and prioritization for personal growth
		Achieving satisfaction and contentment through the mentorship process
		Developing an action plan to achieve set goals
		Tracking progress and outcomes by writing down achievements
		Setting specific time frames for addressing weaknesses and measuring outcomes
		Timeline and measurement of success
	Portfolio & reflection	Submitting and building a portfolio
		Portfolio writing for mentors and mentees
		Measuring the success of subjective goals through portfolios
		Using the portfolio to measure progress and achievements
		Tracking progress and growth through reflective practices
		Holding oneself accountable for achieving set goals
C. Building Positive Mentor-Mentee Relationships:	Mentoring Culture	Ensuring that everyone feels seen, heard, and valued
		Promoting equal opportunities for growth and creating a supportive culture
		Developing positive relationships between mentors and mentees
		Determining the level of trust and depth of the mentor-mentee relationship
		Dealing with challenges and conflicts in the mentor-mentee relationship
		Addressing communication issues and conflicts
		Emphasizing positive analysis and avoiding negativity
		Fostering a culture of helping others and providing equal opportunities for growth
	Qualities and Responsibilities	Being a role model as a mentor and improving mentoring sessions
		Defining/outlining responsibilities of mentors and mentees
		Qualities of effective mentors
		Recognizing and accepting other’s weaknesses
		Being open-minded and comfortable with change
		Importance of integrity and respect in mentoring relationships
	Communication Skills:	Communication Fundamentals of maintaining a mentor-mentee relationship
		Avoiding direct rejection when saying “no” to seniors
		Importance of communication and valuing interactions

The workshop participants highlighted the positive role of formal mentorship program that is owned at the organizational level with defined structure, outcomes and support systems with a special focus on time allocation and faculty training sessions in the domain of mentoring. The special emphasis was given by the participants on their own role as mentors and mentees that can be enhanced through self-awareness and analysis. This awareness was identified as the basis on which the rapport building between mentors and mentees can be improved. the progress and effort can then be tracked through the development of personal development plans leading to actionable outcomes and the analysis of the same through the development of a reflective mentoring portfolio. According to the faculty this training workshop provided them with a clear understanding of the process of development of a mentor mentee relationship and the effectiveness of developing a culture for the same through defining specific roles and responsibilities and a command on the basic communication skills to obtain the desired outcomes. The faculty agreed that this process of mentoring demands a holistic approach where the organization, the faculty and the administration needs to come together to lead to personal and professional developments where self-aware and responsible faculty members can be trained and retained for better student outcomes.

## Discussion

The theoretical framework for faculty development in mentoring is based on the Maslow’s hierarchy of needs pyramid, recognizing that individuals progress towards self-actualization only when their basic needs are fulfilled [23]. The participants in this study highlighted those institutional policies promote a culture and climate for transfer of knowledge, skills, and resources from experienced mentors to their mentees that makes the basic foundation for professional growth and the effective attainment of career goals and this finding corroborates with the available literature on the subject [24].

Day 1 and Day 2 sessions combined didactic and experiential components in alignment with the mentoring workshop programs which have been reported to result in improved mentor-mentee relationship between women graduate students and their science advisors [25]. Same principle in our workshop helped participants self-analyze and develop career development plans, through interaction with peers and facilitators and making maximum use of the hands-on activity. Post workshop evaluation and reflection from the participants revealed that all faculty members; junior, mid-career and senior acquired new knowledge, developed understanding and acquired competencies which align with the results of another study in which mid-and senior-level investigators in lower middle-income counties (LMICs) acquired effective mentoring and developed communication and leadership skills through well designed training interventions [10].

In line with the present findings, skill development literature for being effective mentors and mentees suggests an association of focused mentoring with mentees’ behavior, job satisfaction and affective organizational commitment [26^27^, ^28^]. It is also documented that the provision of resources, protected time, and internal funding is crucial for facilitating the academic growth of mentees and promoting scholarly activities [29], as highlighted by the participants in the present study. These workshops are also reported to develop mentorship culture and with improvement in organizational support can enhance faculty retention and mentee satisfaction [30]. In addition to resources and administrative support, literature also documents rewards, incentives, and recognition of mentors for the sustainability of the program [16] which were highlighted by the study participants.

Mentorship is important for early-career researchers to navigate challenges in academic and nonacademic existence and career development [25]. A study conducted at AKU-MC recommends that capacity building activities and opportunities for the faculty development should be provided to the faculty mentors as well as mentees [16]. The workshop participants highlighted the need to organize mentorship workshops to increase faculty knowledge on mentorship, actual mentorship skills and practices. With reference to mentor mentee dynamics, it was noticed that communication of mentors and mentees for structured meetings with support from administration can contribute to academic performance in higher education settings which again augments other findings from previous similar investigations [31].

Present study participants also emphasized the role of personal development plan in terms of self-reflection, mentorship skills development and leadership capabilities, developing an action plan and managing time wisely along with using portfolio for documenting achievements and measuring progress. In a study conducted by Paige Haber-Curran et al., mentors identified several personal and educational advantages associated with mentorship. They report the mutual benefits that mentors and mentees derive from their mentoring relationships. A significant aspect of mentors’ growth is linked to their personal development and competence, which includes enhancements in diverse areas, such as time management, self-confidence, effective communication, leadership skills, organizational and planning abilities, personal satisfaction and recognition, individual sense of purpose, and the development of introspective and reflective learning practices [32]. It has been previously suggested that mentors can further strengthen their personal development by engaging in workshops that focus on various aspects, including time management. This participation allows mentors to attain a balanced approach in handling responsibilities that come with in mentorship [32].

Mentoring is known to contribute to goal formation and clarification. In a case study involving mentoring within a Teacher Development program in Ankara, Turkey, it was found that mentoring promotes self-evaluation among both mentors and mentees. This process leads to skill enhancements in areas such as communication and reflection by mentees achieved by observing the mentor’s actions. Additionally, mentorship assists in recognizing strengths and priorities, of both mentors and mentees [33]. The faculty involced in self analysis through personal SWOT in this study felt better able to identify what they needed to improve in themselves for ensuring mentor-mentee relationships that have enough value to influence each other and the system.

The identified barriers to mentoring; action plans by individual participants; evaluations and follow-up actions through the post-workshop surveys can help in impactful trainings of mentorship and improve the mentorship practices [10]. Considering the importance of mentorship programs for the individuals and the institutions it is crucial to address existing gaps in mentoring programs, including equitable mentor and mentee recruitment and evaluate the program outcomes to ensure that mentorship experiences are successful and beneficial [34]. Investing resources in mentor training and compensating mentors for their time and efforts is essential to sustain a robust mentoring ecosystem. Finally, continuous evaluation of the outcomes and impact of mentoring efforts should be conducted to ensure their effectiveness and make necessary improvements [35]. These themes were highlighted by the faculty members of medical institutions in the study who reported that the organizational factors are crucial to ensure success, self-evaluation is a necessity for any formal relationship between a mentor and a mentee and having clear and transparent goals with well designed realistic timelines tagged by achievable milestones are the pillars on which institutions of developing world can build their formal mentorships programs.

The present study is focused on the medical faculty already exposed to the concept of faculty mentoring therefore the data collected can have the impact from previous learning as well however approximately all participants were conducting SWOT and making action plans for the first time. Only a group of medical faculties of the institutions were involved in the study which needs to be further expanded to attain more generalizability of the study findings. Study data collected during or post workshop can be influenced by social desirability bias, to mitigate this feedback was anonymized during and after the workshop . Participants may have provided responses they perceived as favorable or expected by the researchers. The present study focused on immediate post-workshop feedback and perceptions which needs to be furthered through designing long term evaluations by the interested researchers in the field.

## Conclusion

Medical Faculty of Pakistani Universities at all career levels is interested in development of formal mentoring programs in their universities. Participants in our study emphasized the role of organization support in terms of provision of resources, guidance, and opportunities for professional development in mentorship programs. The workshops conducted at AKU and UoL recognized the need of organizational policies, need of comprehensive formal training of faculty mentors especially in the dynamics of mentor mentee relationship, self-analysis leading to formulation of personal mentor mentee development plans and having structured mentor and mentee portfolios as evidence of their effort and progress in their journey of personal and professional development through meaningful connections.

By implementing the identified strategies, institutions can cultivate a culture of mentorship that supports the professional growth and success of academics for cultivating the minds that are in turn shaping our future generations. Establishing an evidence-based foundation for mentorship programs, the health professions institutions mayl be able to cultivate a robust mentoring culture that not only bridges the existing gaps but also contributes to the overall professional growth and success of faculty members in the diverse higher education institutions.

Future research needs to validate the key outcome of these workshops in diverse participants from different institutions. This will facilitate in transferring skills of the experienced facilitators to novice faculty members that is required for their professional growth and development. The collaborative initiatives have the potential to set transformative precedent, fostering a culture of mentorship that not only benefits the participating institutions but also serves as a model for elevating the standards of faculty development across the entire higher education landscape in Pakistan.

## Data Availability

Data is provided within the manuscript or supplementary information files.
